# Supplementary figures and images for: Transcriptomic analysis reveals effects of fertilization towards growth and quality of Fritillariae thunbergii bulbus
Source: PLoS One. 2024 Sep 20;19(9):e0309978. doi: 10.1371/journal.pone.0309978 (PMC11414930; doi:10.1371/journal.pone.0309978)

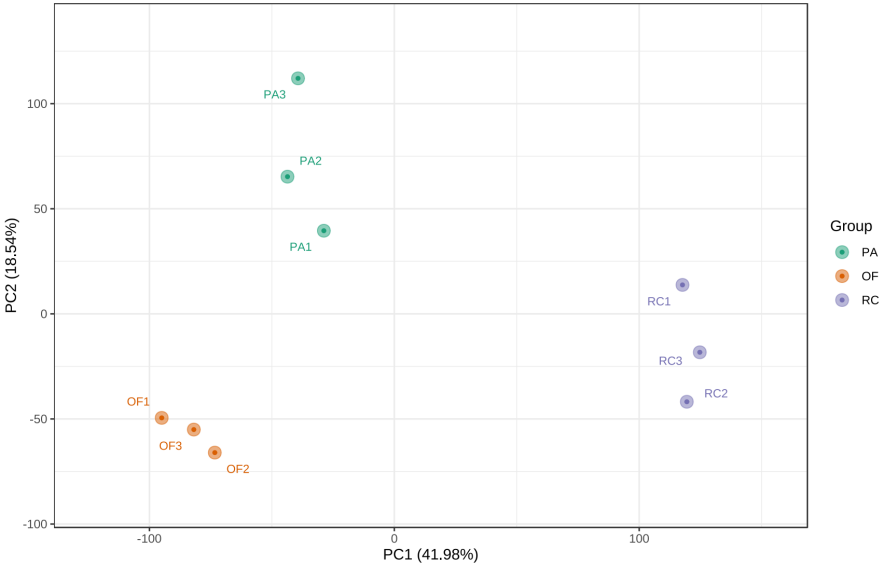

Supplement: S1 Fig — (TIF) [file pone.0309978.s001.tif]

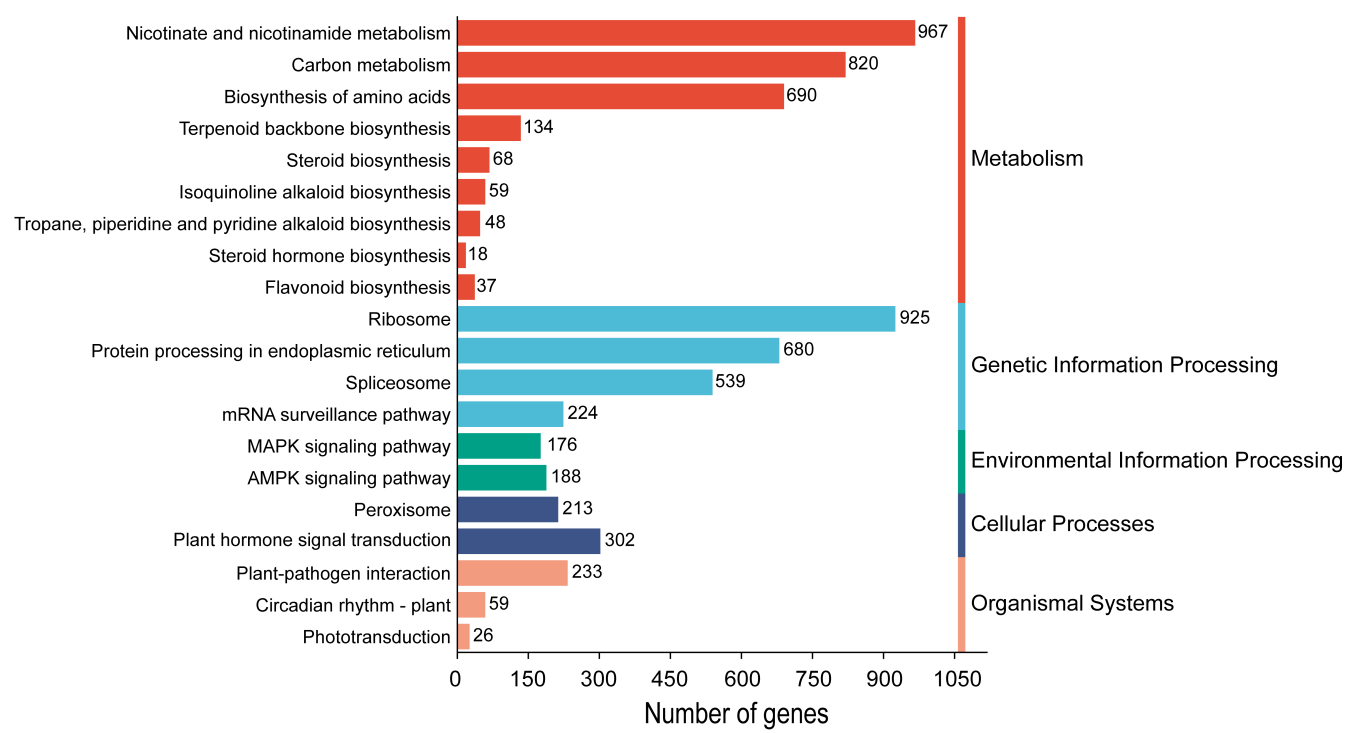

Supplement: S2 Fig — (A) Homologous species distribution unigenes about FTB compared against the Nr database. (B) GO function annotation and classification statistics of the assembled unigenes. The results are summarized in three main categories: BP, biological process; CC, cellular component; MF, molecular function. (C) KEGG function annotation and classification statistics of the assembled unigenes. (ZIP) [file pone.0309978.s002.zip › S2C_Fig.tif]

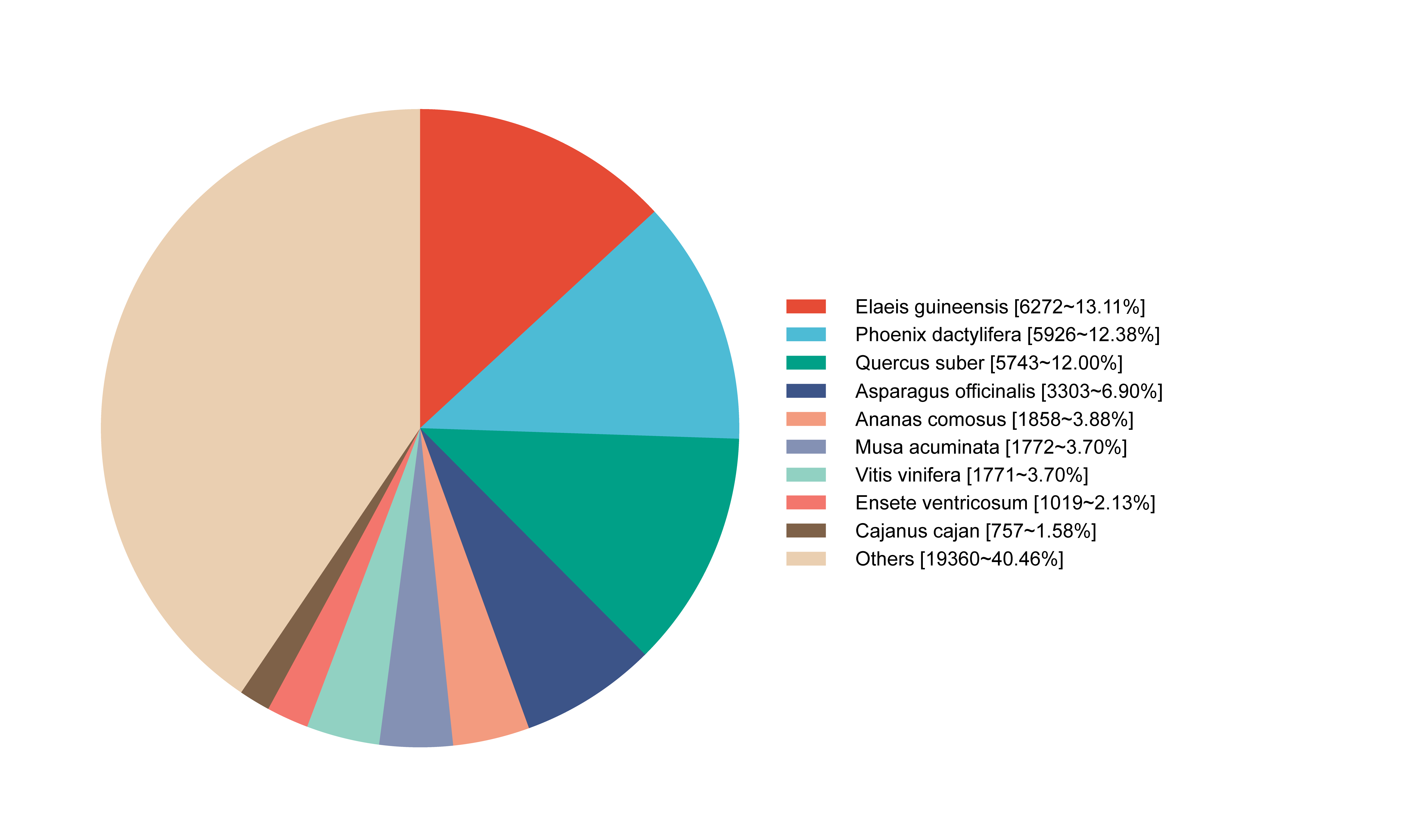

Supplement: S2 Fig — (A) Homologous species distribution unigenes about FTB compared against the Nr database. (B) GO function annotation and classification statistics of the assembled unigenes. The results are summarized in three main categories: BP, biological process; CC, cellular component; MF, molecular function. (C) KEGG function annotation and classification statistics of the assembled unigenes. (ZIP) [file pone.0309978.s002.zip › S2A_Fig.tif]

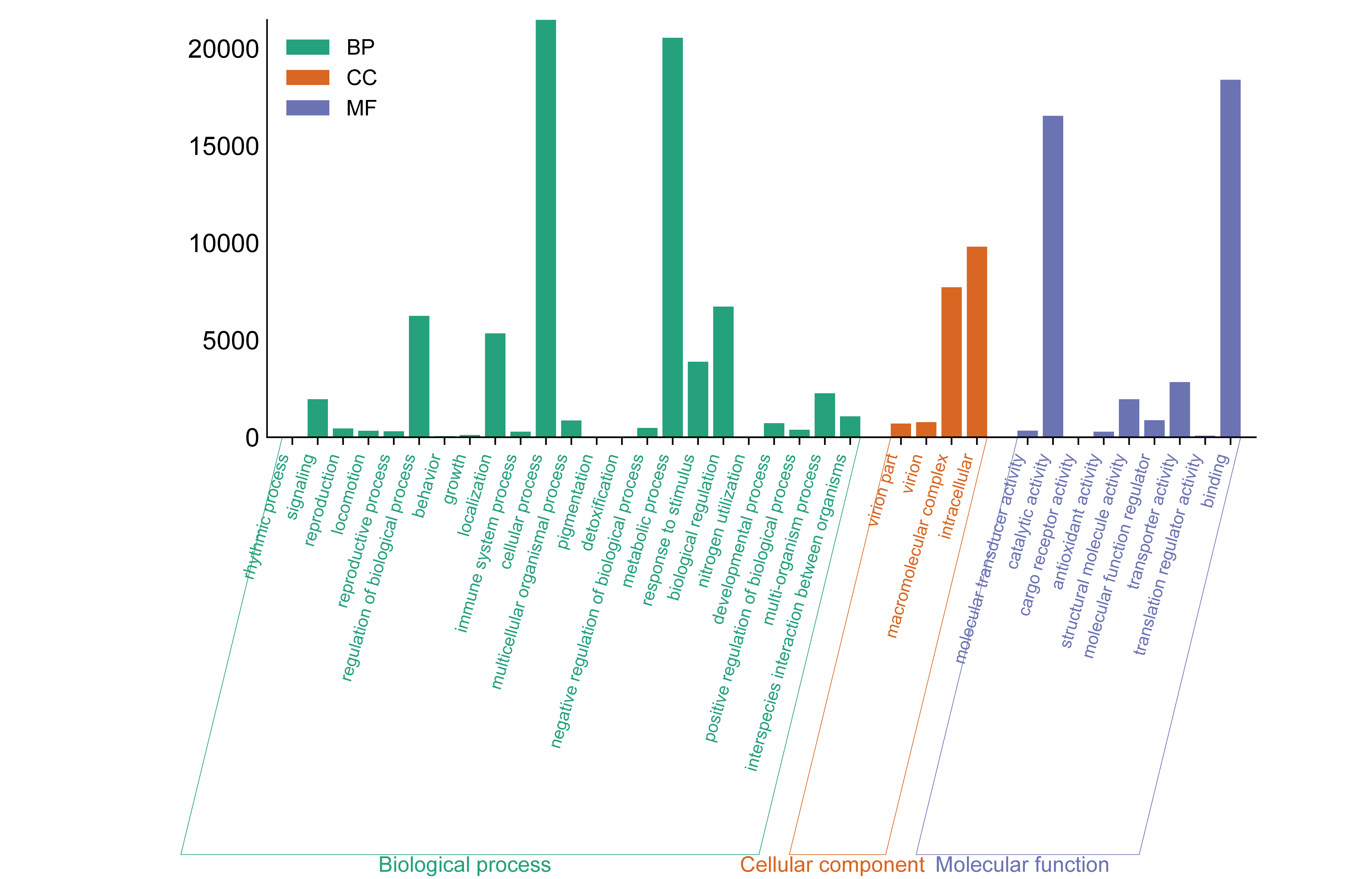

Supplement: S2 Fig — (A) Homologous species distribution unigenes about FTB compared against the Nr database. (B) GO function annotation and classification statistics of the assembled unigenes. The results are summarized in three main categories: BP, biological process; CC, cellular component; MF, molecular function. (C) KEGG function annotation and classification statistics of the assembled unigenes. (ZIP) [file pone.0309978.s002.zip › S2B_Fig.tif]
