# Supplementary material for: Transcriptomic analysis reveals effects of fertilization towards growth and quality of Fritillariae thunbergii bulbus
Source: PLoS One. 2024 Sep 20;19(9):e0309978. doi: 10.1371/journal.pone.0309978 (PMC11414930; doi:10.1371/journal.pone.0309978)
Supplement: S1 Table — (DOCX) [file pone.0309978.s003.docx]

**S1 Table. Primers for qRT-PCR analysis.**

| Number | Name | Gene ID | Forward primer (5' to 3') | Reverse primer (5' to 3') |
| --- | --- | --- | --- | --- |
| 1 | *Actin* | — | CATAATCCAGAGCCACATA | TGCCAATCTACGAGGGTT |
| 2 | *PP2C* | Cluster-73431.7500 | CATAATCCAGAGCCACATA | TGCCAATCTACGAGGGTT |
| 3 | *SnRK2* | Cluster-73431.37475 | GTTACGGGAGTGGGAGACGA | AGTCTCATCACCTCGGCACA |
| 4 | *ABF* | Cluster-73431.17038 | TCGAGGTCAATCGGTTCGGA | CTCTACGGCCTCACCCTCAA |
| 5 | *JAZ* | Cluster-73431.43172 | CAGCCCATGGACCTGTTTCC | TTGTCTGCCGGAAAGTCGTG |
| 6 | *ACAT* | Cluster-73431.30459 | TGGCAAGATCGGAGTCGCTA | CGGGAAAGGCCTGCACATTA |
| 7 | *MVD* | Cluster-73431.14145 | GCATCTGCTTGGGAACTGCT | GGCCGACAGCAAGATCGTAG |
| 8 | *DXS* | Cluster-73431.16427 | GCAAGGCCCTCGTCAAACTT | GGTCACGTTCTTAGCGGCTT |
| 9 | *FPS* | Cluster-73431.41251 | CCCTCCCTCTTCTGCTCCTC | GCGGTGACGATGCTAGGTTT |
| 10 | *NSDHL* | Cluster-73431.16347 | GTTGTGTCCCTGCAGGATGG | AGCAACTGTCCCACTGCCTA |
| 11 | *CYP94N1* | Cluster-73431.14169 | CGGAGGTGACGCAAAGCATA | GCAGCACATCATCCACCACA |
| 12 | *CYP90G1* | Cluster-73431.29395 | CGGTTGTCTCCTCCATCCCT | TCGCCCATTGGAGCTGTTTC |
| 13 | *GABAT1* | Cluster-73431.19556 | GGCCCGCATACTCTTAAGGC | CGCACCACCGAATTCTGACA |
