# Supplementary material for: Transcriptomic analysis reveals effects of fertilization towards growth and quality of Fritillariae thunbergii bulbus
Source: PLoS One. 2024 Sep 20;19(9):e0309978. doi: 10.1371/journal.pone.0309978 (PMC11414930; doi:10.1371/journal.pone.0309978)
Supplement: S2 Table — (DOCX) [file pone.0309978.s004.docx]

**S2 Table. Numbers and quality of RNA-seq reads produced in each sample.**

| Sample | Raw Reads | Clean Reads | Q20(%) | Q30(%) | GC Content (%) |
| --- | --- | --- | --- | --- | --- |
| PARC_1 | 20876500 | 20699578 | 97.46 | 92.89 | 48.13 |
| PARC_2 | 23445564 | 23265345 | 97.38 | 92.68 | 47.86 |
| PARC_3 | 22795626 | 22546168 | 97.14 | 92.22 | 47.86 |
| PASF_1 | 20259466 | 20136059 | 97.3 | 92.68 | 50.93 |
| PASF_2 | 21252493 | 21149932 | 97.31 | 92.71 | 50.64 |
| PASF_3 | 23133567 | 22979500 | 96.77 | 91.78 | 50.82 |
| PALS_1 | 21275044 | 21150078 | 97.23 | 92.51 | 50.2 |
| PALS_2 | 22251905 | 22063146 | 97.22 | 92.52 | 50.56 |
| PALS_3 | 21844033 | 21680130 | 96.91 | 91.9 | 49.68 |
