# Supplementary material for: Transcriptomic analysis reveals effects of fertilization towards growth and quality of Fritillariae thunbergii bulbus
Source: PLoS One. 2024 Sep 20;19(9):e0309978. doi: 10.1371/journal.pone.0309978 (PMC11414930; doi:10.1371/journal.pone.0309978)
Supplement: S3 Table — (DOCX) [file pone.0309978.s005.docx]

**S3 Table. Length distribution of unigenes from the transcriptome data.**

| Length | Unigenes | |
| --- | --- | --- |
| distribution | Number | Percentage％ |
| 300-500bp | 48667 | 42.24% |
| 500-1000bp | 40133 | 34.83% |
| 1000-2000bp | 17981 | 15.61% |
| >2000bp | 8644 | 7.50% |
| Total | 115224 |  |
