# Supplementary material for: Transcriptomic analysis reveals effects of fertilization towards growth and quality of Fritillariae thunbergii bulbus
Source: PLoS One. 2024 Sep 20;19(9):e0309978. doi: 10.1371/journal.pone.0309978 (PMC11414930; doi:10.1371/journal.pone.0309978)
Supplement: S4 Table — (DOCX) [file pone.0309978.s006.docx]

**S4 Table. Transcripts and FPKM of genes involved in plant hormone signal transduction (ko04075).**

| Number | Name | Gene ID | FPKM | | |
| --- | --- | --- | --- | --- | --- |
|  |  |  | RC | OF | PA |
| 1 | AUX1 | Cluster-73431.25816 | 2.68 | 0.64 | 0.466666667 |
| 2 | TIR1 | Cluster-73431.44640 | 9.07 | 18.32333333 | 14.49 |
|  |  | Cluster-73431.73431.46990 | 0.160003333 | 0.796666667 | 0.873333333 |
| 3 | IAA | Cluster-73431.30776 | 1.843333333 | 0.06334 | 0.076673333 |
|  |  | Cluster-73431.32254 | 38.98666667 | 121.5766667 | 65.03666667 |
|  |  | Cluster-73431.34219 | 129.0533333 | 378.8133333 | 262.8566667 |
|  |  | Cluster-73431.27704 | 43.95 | 20.15666667 | 21.14333333 |
|  |  | Cluster-73431.29846 | 25.05 | 9.696666667 | 10.64 |
|  |  | Cluster-73431.42024 | 10.31 | 7.92 | 12.66666667 |
|  |  | Cluster-73431.27190 | 22.70666667 | 28.80333333 | 35.21333333 |
| 4 | ARF | Cluster-73431.36017 | 25.3 | 31.27 | 26.85333333 |
| 5 | GH3 | Cluster-73431.10485 | 5.316666667 | 1.703333333 | 6.643333333 |
|  |  | Cluster-73431.41309 | 21.57 | 6.123333333 | 32.18333333 |
|  |  | Cluster-73431.14586 | 5.04 | 9.103333333 | 38.91666667 |
| 6 | SAUR | Cluster-18760.0 | 0.06334 | 2.49 | 2.863333333 |
|  |  | Cluster-73431.8112 | 8.076666667 | 3.876666667 | 11.42333333 |
|  |  | Cluster-73431.15747 | 11.09666667 | 2.08 | 8.756666667 |
|  |  | Cluster-73431.43300 | 30.63333333 | 47.87666667 | 44.72333333 |
|  |  | Cluster-73431.4758 | 7.993333333 | 1.833333333 | 1.823333333 |
|  |  | Cluster-17882.0 | 0.903336667 | 0.466666667 | 2.436666667 |
| 7 | CRE1 | Cluster-73431.40564 | 16.49 | 9.32 | 12.83333333 |
|  |  | Cluster-73431.33444 | 22.51 | 14.79666667 | 16.09333333 |
|  |  | Cluster-73431.24162 | 17.81666667 | 7.263333333 | 4.056666667 |
|  |  | Cluster-73431.24161 | 7.533333333 | 1.003333333 | 0.543336667 |
|  |  | Cluster-73431.24168 | 10.74 | 3.15 | 2.896666667 |
|  |  | Cluster-73431.27484 | 7.22 | 4.053333333 | 0.353336667 |
|  |  | Cluster-73431.24163 | 12.80333333 | 9.116666667 | 11.39666667 |
| 8 | AHP | Cluster-73431.41993 | 9.49 | 33.96333333 | 28.08 |
|  |  | Cluster-55427.0 | 24.00666667 | 0.580003333 | 1.600003333 |
| 9 | ARR-B | Cluster-44657.0 | 0.00001 | 0.220003333 | 0.553333333 |
|  |  | Cluster-64510.0 | 0.376666667 | 0.483333333 | 0.886666667 |
|  |  | Cluster-73431.46971 | 7.723333333 | 3.463333333 | 3.283333333 |
|  |  | Cluster-73431.44552 | 5.336666667 | 2.95 | 4.653333333 |
|  |  | Cluster-73431.18823 | 9.766666667 | 14.32333333 | 19.46666667 |
|  |  | Cluster-73431.6522 | 0.7 | 0.110003333 | 0.00001 |
|  |  | Cluster-73431.37071 | 4.373333333 | 1.33 | 1.16 |
| 10 | ARR-A | Cluster-73431.39293 | 18.16666667 | 1.976666667 | 5.216666667 |
|  |  | Cluster-73431.27790 | 25.41333333 | 15.01666667 | 19.08 |
|  |  | Cluster-73431.23265 | 14.4 | 4.523333333 | 9.613333333 |
| 11 | GID1 | Cluster-73431.18267 | 31.62666667 | 15.54 | 44.68333333 |
|  |  | Cluster-73431.33419 | 45.01333333 | 51.86333333 | 78.46666667 |
| 12 | GID2, SLY1 | Cluster-73431.19882 | 1.096666667 | 0.55 | 0.080003333 |
|  |  | Cluster-73431.35464 | 15.74 | 42.24333333 | 23.41 |
|  |  | Cluster-73431.8128 | 9.286666667 | 23.13666667 | 12.78666667 |
| 13 | DELLA | Cluster-73431.33582 | 8.243333333 | 21.83666667 | 24.08 |
|  |  | Cluster-73431.27471 | 105.7233333 | 84.36 | 80.80666667 |
| 14 | TF | Cluster-74546.0 | 0.00001 | 0.00001 | 0.513333333 |
| 15 | PYL | Cluster-23563.0 | 0.00001 | 2.566666667 | 3.283333333 |
|  |  | Cluster-73431.31192 | 23.68333333 | 34.83666667 | 43.74333333 |
|  |  | Cluster-73431.20709 | 15.46333333 | 33.79 | 23.7 |
|  |  | Cluster-64830.0 | 1.900006667 | 1.756666667 | 11.64 |
|  |  | Cluster-73431.318 | 0.606666667 | 15.61 | 7.81 |
|  |  | Cluster-73431.28650 | 3.806666667 | 7.486666667 | 6.426666667 |
|  |  | Cluster-73431.2267 | 0.00001 | 5.19 | 0.633336667 |
| 16 | PP2C | Cluster-73431.30921 | 8.836666667 | 14.47 | 9.593333333 |
|  |  | Cluster-73431.39507 | 28.83 | 10.28666667 | 28.8 |
|  |  | Cluster-73431.750 | 0.17 | 2.55 | 5.006666667 |
| 17 | SNRK2 | Cluster-73431.31896 | 56.79666667 | 20.86666667 | 18.34333333 |
|  |  | Cluster-73431.27456 | 0.53667 | 1.78 | 1.9 |
|  |  | Cluster-73431.27622 | 16.00333333 | 15.86666667 | 19.48333333 |
|  |  | Cluster-73431.37475 | 8.35 | 12.65 | 18.48666667 |
|  |  | Cluster-73431.25981 | 10.47 | 16.61666667 | 18.23333333 |
|  | ABF | Cluster-73431.11663 | 0.53667 | 1.9 | 1.78 |
|  |  | Cluster-73431.14153 | 8.35 | 18.48666667 | 12.65 |
|  |  | Cluster-73431.17038 | 10.47 | 18.23333333 | 16.61666667 |
| 19 | ETR, ERS | Cluster-73431.31271 | 8.633333333 | 22.81333333 | 26.90333333 |
|  |  | Cluster-73431.25240 | 9.693333333 | 76.91333333 | 70.17666667 |
|  |  | Cluster-73431.30690 | 55.99666667 | 122.8266667 | 99.22333333 |
| 20 | CTR1 | Cluster-73431.36126 | 22.92666667 | 27.83666667 | 22.16666667 |
| 21 | MPK6 | Cluster-73431.30157 | 89.32666667 | 68.48666667 | 76.37666667 |
|  |  | Cluster-41970.0 | 1.453333333 | 0.336666667 | 0.616666667 |
| 22 | EIN2 | Cluster-73431.39230 | 3.253333333 | 1.21 | 1.183333333 |
| 23 | BKI1 | Cluster-73431.7400 | 2.57 | 0.7 | 1.02 |
| 24 | BSK | Cluster-73431.34177 | 38.71 | 21.06666667 | 23.65666667 |
|  |  | Cluster-73431.34995 | 63.27666667 | 52.38333333 | 53.10333333 |
| 25 | BZR1_2 | Cluster-73431.35716 | 21.22666667 | 48.85333333 | 54.85 |
| 26 | JAR1 | Cluster-73431.32731 | 78.97 | 279.1633333 | 213.9966667 |
| 27 | COI-1 | Cluster-73431.33118 | 34.18333333 | 43.65 | 29.95 |
| 28 | JAZ | Cluster-73431.41447 | 10.60333333 | 7.446666667 | 25.39333333 |
|  |  | Cluster-73431.43371 | 86.85 | 42.81 | 154.04 |
|  |  | Cluster-73431.43172 | 7.43 | 20.35666667 | 47.48 |
|  |  | Cluster-73431.16410 | 6.28 | 46.25333333 | 46.68333333 |
|  |  | Cluster-73431.16412 | 10.67 | 60.24 | 77.16666667 |
|  |  | Cluster-73431.36863 | 7.263333333 | 2.263333333 | 9.94 |
|  |  | Cluster-73431.36864 | 66.88 | 84.13333333 | 100.3433333 |
|  |  | Cluster-73431.26241 | 61.88666667 | 29.95333333 | 114.0633333 |
|  |  | Cluster-73431.50647 | 0.00001 | 9.796666667 | 4.136666667 |
|  |  | Cluster-58316.0 | 4.69 | 1.103333333 | 4.09 |
|  |  | Cluster-73431.29779 | 60.59333333 | 14.37666667 | 62.91333333 |
|  |  | Cluster-73431.14882 | 4.866666667 | 3.013333333 | 10.35666667 |
|  |  | Cluster-73431.14881 | 48.98666667 | 17.08333333 | 62.84666667 |
|  |  | Cluster-73431.50648 | 0.00001 | 41.13666667 | 11.04 |
|  |  | Cluster-73431.40838 | 70 | 42.25666667 | 136.72 |
|  |  | Cluster-73431.27178 | 188.65 | 157.0766667 | 262.8733333 |
|  |  | Cluster-73431.41887 | 50.66 | 26.76666667 | 54.49666667 |
|  |  | Cluster-73431.27177 | 21.23 | 5.223333333 | 8.28 |
|  |  | Cluster-73431.15810 | 19.74666667 | 69.96666667 | 65.79 |
|  |  | Cluster-73431.44542 | 9.27 | 1.676666667 | 9.463333333 |
|  |  | Cluster-73431.25669 | 202.2866667 | 58.06333333 | 264.3666667 |
|  |  | Cluster-73431.29865 | 284.9933333 | 137.7733333 | 409.0966667 |
|  |  | Cluster-73431.26238 | 198.1533333 | 106.62 | 382.2433333 |
|  |  | Cluster-73431.13653 | 1.510003333 | 17.41666667 | 16.67666667 |
|  |  | Cluster-73431.29130 | 7.233333333 | 244.2733333 | 161.2766667 |
|  |  | Cluster-73431.13531 | 40.90666667 | 7.573333333 | 39.05333333 |
|  |  | Cluster-73431.36857 | 11.29 | 2.853333333 | 4.496666667 |
|  |  | Cluster-73431.12522 | 4.523333333 | 2.46 | 12.86 |
|  |  | Cluster-73431.13881 | 2.356666667 | 13.90666667 | 7.62 |
| 29 | MYC2 | Cluster-73431.31918 | 109.89 | 314.39 | 250.9833333 |
| 30 | NPR1 | Cluster-73431.14445 | 3.043333333 | 14.61333333 | 10.27 |
|  |  | Cluster-73431.24772 | 19.94 | 9.35 | 13.09666667 |
|  |  | Cluster-73431.29525 | 15.63666667 | 41.90666667 | 28.47333333 |
| 31 | TGA | Cluster-73431.40901 | 19.82666667 | 14.00333333 | 11.17 |
|  |  | Cluster-73431.32196 | 14.18333333 | 6.87 | 10.75333333 |
|  |  | Cluster-73431.34379 | 3.246666667 | 0.18667 | 0.510003333 |
|  |  | Cluster-73431.34719 | 39.73666667 | 36.45666667 | 33.24666667 |
