# Supplementary material for: Transcriptomic analysis reveals effects of fertilization towards growth and quality of Fritillariae thunbergii bulbus
Source: PLoS One. 2024 Sep 20;19(9):e0309978. doi: 10.1371/journal.pone.0309978 (PMC11414930; doi:10.1371/journal.pone.0309978)
Supplement: S5 Table — (DOCX) [file pone.0309978.s007.docx]

**S5 Table. Transcripts and FPKM of genes involved in plant-pathogen interaction (ko04626).**

| Number | Name | Gene ID | FPKM | | |
| --- | --- | --- | --- | --- | --- |
|  |  |  | RC | OF | PA |
| 1 | CDPK | Cluster-49922.0 | 0.71 | 0.26 | 0.193336667 |
|  |  | Cluster-73431.43452 | 32.26 | 46.08 | 45.51 |
|  |  | Cluster-65485.0 | 1.533333333 | 0.20667 | 0.00001 |
|  |  | Cluster-73431.34865 | 18.42 | 24.39 | 23.73333333 |
|  |  | Cluster-73431.21079 | 9.283333333 | 4.37 | 6.09 |
|  |  | Cluster-73431.21083 | 18.79333333 | 38.60333333 | 25.71666667 |
|  |  | Cluster-73431.21082 | 3.24 | 0.616666667 | 1.243333333 |
|  |  | Cluster-73431.26402 | 50.51666667 | 71.81333333 | 69.03 |
|  |  | Cluster-73431.15097 | 1.3 | 0.543333333 | 0.08334 |
|  |  | Cluster-73431.31193 | 30.28 | 4.413333333 | 24.48 |
|  |  | Cluster-73431.33664 | 10.16 | 0.556666667 | 4.403333333 |
|  |  | Cluster-73431.26403 | 45.74666667 | 104.43 | 82.19666667 |
|  |  | Cluster-73431.25151 | 25.35333333 | 4.91 | 19.39333333 |
|  |  | Cluster-73431.14226 | 15.76333333 | 23.21 | 21.76666667 |
|  |  | Cluster-73431.31633 | 218.68 | 188.41 | 175.9833333 |
|  |  | Cluster-73431.27692 | 9.573333333 | 17.45333333 | 12.07 |
|  |  | Cluster-73431.15858 | 1.14 | 0.060003333 | 0.400003333 |
|  |  | Cluster-73431.15857 | 13.55333333 | 56.24333333 | 36.72666667 |
|  |  | Cluster-73431.15856 | 10.11666667 | 25.38 | 14.14333333 |
| 2 | RBOH | Cluster-73431.35721 | 6.78 | 2.123333333 | 5.073333333 |
|  |  | Cluster-73431.35720 | 68.18333333 | 20.50333333 | 77.82666667 |
|  |  | Cluster-73431.33519 | 67.6 | 31.29333333 | 89.65 |
|  |  | Cluster-73431.30041 | 4.893333333 | 10.14 | 14.43333333 |
|  |  | Cluster-73431.17965 | 10.67 | 4.963333333 | 8.1 |
| 3 | CNGC | Cluster-73431.31029 | 25.70666667 | 36.19333333 | 30.22333333 |
|  |  | Cluster-73431.14355 | 0.796666667 | 0.333333333 | 0.16334 |
|  |  | Cluster-73431.38574 | 3.926666667 | 0.433333333 | 0.273336667 |
|  |  | Cluster-73431.24348 | 26.87333333 | 17.05333333 | 16.02666667 |
|  |  | Cluster-47823.0 | 1.323333333 | 0.753333333 | 0.243333333 |
| 4 | CALM | Cluster-73431.13320 | 13.41 | 5.086666667 | 10.22333333 |
|  |  | Cluster-73431.16846 | 28.77333333 | 39.37333333 | 61.98333333 |
|  |  | Cluster-73431.33960 | 4.613333333 | 0.973333333 | 0.853336667 |
|  |  | Cluster-73431.37296 | 10.56666667 | 3.443333333 | 3.003333333 |
|  |  | Cluster-73431.25954 | 57.67333333 | 131.1533333 | 105.34 |
|  |  | Cluster-73431.25953 | 27.94666667 | 67.23666667 | 64.17333333 |
| 5 | CML | Cluster-73431.9886 | 9.026666667 | 29.3 | 22.83 |
|  |  | Cluster-73431.12633 | 101.21 | 29.41333333 | 84.01333333 |
|  |  | Cluster-73431.30166 | 159.3733333 | 265.0666667 | 302.2833333 |
|  |  | Cluster-73431.18554 | 16.39333333 | 7.303333333 | 42.89666667 |
|  |  | Cluster-73431.43153 | 71.33 | 18.44666667 | 70.43 |
|  |  | Cluster-73431.24838 | 25.36 | 37.31333333 | 23.16333333 |
|  |  | Cluster-73431.28473 | 65.66333333 | 216.5033333 | 123.8 |
|  |  | Cluster-73431.16361 | 22.18666667 | 14.07666667 | 23.47666667 |
|  |  | Cluster-73431.21353 | 5.866666667 | 0.156673333 | 0.603336667 |
|  |  | Cluster-73431.18668 | 42.24666667 | 19.56666667 | 47.77666667 |
|  |  | Cluster-73431.50076 | 4.473333333 | 6.653333333 | 22.74 |
|  |  | Cluster-73431.33416 | 2.996666667 | 4.003333333 | 14.79 |
|  |  | Cluster-73431.28007 | 52.81333333 | 66.59 | 95.24 |
|  |  | Cluster-73431.30719 | 24.15 | 5.713333333 | 13.48666667 |
|  |  | Cluster-73431.1024 | 0.00001 | 0.096673333 | 3.923333333 |
|  |  | Cluster-73431.27192 | 4.713333333 | 0.25 | 1.53 |
|  |  | Cluster-73431.12968 | 14.29333333 | 5.626666667 | 19.03333333 |
|  |  | Cluster-73431.17722 | 108.9833333 | 81.14666667 | 87.94666667 |
|  |  | Cluster-73431.27184 | 37.57666667 | 19.99 | 36.20666667 |
|  |  | Cluster-73431.45243 | 2.286666667 | 2.39 | 4.99 |
|  |  | Cluster-73431.32270 | 16.31 | 4.326666667 | 8.983333333 |
|  |  | Cluster-73431.22795 | 15.14333333 | 25.07333333 | 20.23333333 |
| 6 | WRKY33 | Cluster-73431.13138 | 15.43333333 | 7.586666667 | 17.78 |
|  |  | Cluster-73431.35566 | 111.13 | 49.99666667 | 103.0766667 |
| 7 | MEKK1 | Cluster-73431.9920 | 13.30666667 | 14.64666667 | 19.09666667 |
| 8 | MAP2K1 | Cluster-73431.37251 | 131.9766667 | 91.53 | 110.7733333 |
| 9 | MKK4/5 | Cluster-73431.28322 | 33.90333333 | 56.77 | 59.51333333 |
| 10 | WRKY22 | Cluster-73431.25036 | 10.23333333 | 2.526666667 | 18.06 |
| 11 | PTI1 | Cluster-73431.32987 | 55.55 | 36.86333333 | 44.37666667 |
|  |  | Cluster-73431.36409 | 4.003333333 | 1.446666667 | 1.876666667 |
|  |  | Cluster-73431.36410 | 4.92 | 2.64 | 3.143333333 |
|  |  | Cluster-73431.29417 | 55.45333333 | 75.08 | 67.03333333 |
| 12 | CERK1 | Cluster-73431.14458 | 16.91333333 | 29.95 | 21.83 |
| 13 | RPM1 | Cluster-73431.22784 | 23.51333333 | 19.41666667 | 33.85666667 |
| 14 | RIN4 | Cluster-73431.38794 | 3.736666667 | 1.083333333 | 2.486666667 |
| 15 | PBS1 | Cluster-73431.24477 | 20.38666667 | 11.07666667 | 17.78 |
| 16 | SUGT1 | Cluster-73431.18126 | 4.796666667 | 10.9 | 8.223333333 |
| 17 | WRKY2 | Cluster-73431.31528 | 45.99 | 31.16333333 | 29.58666667 |
|  |  | Cluster-73431.31208 | 8.88 | 5.623333333 | 4.836666667 |
| 18 | HSP90A, htpG | Cluster-73431.32412 | 360 | 1027.026667 | 881.9833333 |
|  |  | Cluster-73431.32409 | 30.17 | 137.21 | 122.48 |
|  |  | Cluster-73431.28809 | 65.27333333 | 236.72 | 177.5166667 |
|  |  | Cluster-73431.27900 | 37.82333333 | 159.3333333 | 103.1866667 |
|  |  | Cluster-73431.23263 | 31.76333333 | 56.18 | 41.24666667 |
|  |  | Cluster-73431.32011 | 822.3433333 | 1040.86 | 828.2533333 |
|  |  | Cluster-73431.29933 | 327.62 | 668.1666667 | 445.2666667 |
|  |  | Cluster-73431.20980 | 10.83333333 | 0.443333333 | 4.636666667 |
|  |  | Cluster-60959.0 | 1.84 | 0.4 | 0.73 |
| 19 | PBS1 | Cluster-73431.24477 | 20.38666667 | 11.07666667 | 17.78 |
| 20 | PTI6 | Cluster-73431.40891 | 69.65 | 78.17333333 | 90.37666667 |
| 21 | HSP90B, TRA1 | Cluster-73431.31397 | 155.9066667 | 371.9766667 | 304.79 |
|  |  | Cluster-73431.30558 | 276.93 | 605.4666667 | 531.15 |
