# Supplementary material for: Transcriptomic analysis reveals effects of fertilization towards growth and quality of Fritillariae thunbergii bulbus
Source: PLoS One. 2024 Sep 20;19(9):e0309978. doi: 10.1371/journal.pone.0309978 (PMC11414930; doi:10.1371/journal.pone.0309978)
Supplement: S6 Table — (DOCX) [file pone.0309978.s008.docx]

**S6 Table. Transcripts and FPKM of genes involved in mRNA surveillance pathway (ko03015).**

| Number | Name | Gene ID | FPKM | | |
| --- | --- | --- | --- | --- | --- |
|  |  |  | RC | OF | PA |
| 1 | NCBP1 | Cluster-73431.29667 | 28.51 | 43.9 | 33.94333333 |
|  |  | Cluster-73431.21408 | 1.12 | 0.333333333 | 0.236666667 |
| 2 | RBM8A | Cluster-73431.28432 | 31.2 | 42.19333333 | 35.17666667 |
| 3 | MAGOH | Cluster-73431.23634 | 104.02 | 196.5766667 | 146.7633333 |
| 4 | EIF4A3 | Cluster-73431.47196 | 3.82 | 0.190006667 | 1.166666667 |
|  |  | Cluster-73431.30302 | 84.22333333 | 120.8 | 90.68 |
| 5 | PNN | Cluster-73431.22516 | 28.57 | 26.97 | 17.29333333 |
| 6 | ACIN1 | Cluster-73431.38577 | 18.17333333 | 15.44 | 10.83 |
| 7 | RNPS1 | Cluster-73431.30176 | 22.91 | 41.52666667 | 28.37333333 |
| 8 | THOC4 | Cluster-73431.50371 | 5.36 | 15.37333333 | 4.223333333 |
|  |  | Cluster-73431.30543 | 112.16 | 63.17333333 | 86.17333333 |
| 9 | DDX39B | Cluster-73431.26937 | 0.91 | 0.343333333 | 0.150003333 |
|  |  | Cluster-73431.26935 | 101.09 | 44.52666667 | 56.68 |
| 10 | WIBG | Cluster-73431.17990 | 11.35 | 17.97666667 | 19.09666667 |
| 11 | SRRM1 | Cluster-73431.29844 | 69.7 | 30.42666667 | 25.1 |
|  |  | Cluster-73431.38963 | 12.67 | 6.366666667 | 6.373333333 |
| 12 | RNMT | Cluster-73431.38564 | 25.10666667 | 23.14333333 | 20.3 |
| 13 | PABPN1 | Cluster-73431.26039 | 3.08 | 5 | 3.606666667 |
|  |  | Cluster-73431.13507 | 24.24 | 48.99 | 41.91 |
| 14 | CPSF6/7 | Cluster-73431.12234 | 19.81333333 | 14.3 | 15.31 |
|  |  | Cluster-73431.15546 | 9.916666667 | 5.266666667 | 5.746666667 |
| 15 | PAP | Cluster-73431.19474 | 32.13 | 12.73666667 | 35.26 |
| 16 | CLP1 | Cluster-73431.27158 | 23.33666667 | 22.17666667 | 16.89666667 |
| 17 | PCF11 | Cluster-73431.28719 | 38.6 | 25.64 | 22.53 |
| 18 | CPSF1 | Cluster-73431.21549 | 9.583333333 | 5.42 | 5.443333333 |
| 19 | CPSF4 | Cluster-73431.32307 | 4.413333333 | 13.92 | 14.52333333 |
| 20 | CPSF2 | Cluster-73431.27808 | 28.12666667 | 19.86 | 17.42 |
| 21 | PPP1C | Cluster-73431.18854 | 3.81 | 11.29666667 | 6.536666667 |
|  |  | Cluster-73431.42275 | 2.843333333 | 1.146666667 | 1.506666667 |
|  |  | Cluster-73431.20732 | 124.5433333 | 71.86666667 | 89.46333333 |
| 22 | SSU72 | Cluster-73431.19856 | 1.54 | 3.363333333 | 5.03 |
| 23 | CSTF3 | Cluster-73431.27829 | 33.14666667 | 20.22 | 19.76 |
|  |  | Cluster-73431.21627 | 4.493333333 | 15.34666667 | 11.63333333 |
| 24 | SYMPK | Cluster-62130.0 | 1.163333333 | 0.18 | 0.480003333 |
|  |  | Cluster-73431.38519 | 17.52333333 | 10.43666667 | 10.77333333 |
|  |  | Cluster-73431.25799 | 16.16333333 | 27.24 | 21.67 |
|  |  | Cluster-73431.28721 | 12.95333333 | 10.78333333 | 9.253333333 |
| 25 | MSI | Cluster-73431.22819 | 34.88 | 66.85333333 | 40.63333333 |
|  |  | Cluster-73431.40999 | 34.95 | 66.58666667 | 49.07 |
|  |  | Cluster-73431.36134 | 62.46666667 | 90.14666667 | 66.95 |
|  |  | Cluster-73431.36760 | 42.07333333 | 31.22333333 | 31.99333333 |
|  |  | Cluster-73431.42308 | 8.423333333 | 11.68 | 24.03666667 |
|  |  | Cluster-73431.16752 | 6.443333333 | 18.93 | 15.26666667 |
| 26 | PABPC | Cluster-73431.31717 | 153.36 | 152.1333333 | 135.5166667 |
|  |  | Cluster-73431.26761 | 7.85 | 16.3 | 9.766666667 |
|  |  | Cluster-73431.32224 | 112.17 | 45.78333333 | 54.79666667 |
|  |  | Cluster-73431.25572 | 112.4733333 | 111.8066667 | 81.08333333 |
|  |  | Cluster-73431.25573 | 146.0833333 | 269.31 | 181.4733333 |
|  |  | Cluster-73431.11771 | 16.73666667 | 29.11666667 | 21.33 |
|  |  | Cluster-73431.21565 | 2.553333333 | 0.923333333 | 0.873333333 |
|  |  | Cluster-73431.11960 | 0.676666667 | 0.763333333 | 3.286666667 |
|  |  | Cluster-73431.12158 | 13.78 | 17.75 | 13.91333333 |
| 27 | ETF1 | Cluster-73431.27346 | 272.18 | 185.2 | 173.1033333 |
| 28 | ERF3 | Cluster-73431.28185 | 128.9066667 | 134.21 | 111.0566667 |
| 29 | UPF1 | Cluster-73431.22423 | 1.13 | 1.463333333 | 0.573333333 |
|  |  | Cluster-73431.36744 | 6.91 | 10.57 | 8.02 |
|  |  | Cluster-73431.36745 | 4.123333333 | 3.5 | 1.953333333 |
|  |  | Cluster-73431.36487 | 23.94333333 | 52.20666667 | 23.94333333 |
| 30 | SMG1 | Cluster-73431.27531 | 6.59 | 4.706666667 | 3.296666667 |
| 31 | SMG7 | Cluster-73431.28524 | 53.41 | 29.2 | 33.08666667 |
|  |  | Cluster-73431.32650 | 25.07 | 17.18666667 | 16.58666667 |
| 32 | PPP2C | Cluster-73431.27879 | 29.49333333 | 44.68 | 36.14666667 |
|  |  | Cluster-73431.22822 | 56.31 | 90.45666667 | 69.95 |
|  |  | Cluster-73431.28730 | 109.08 | 78.93 | 88.54666667 |
| 33 | PPP2R1 | Cluster-73431.26161 | 93.16333333 | 124.5166667 | 104.5533333 |
|  |  | Cluster-73431.31813 | 164.4366667 | 105.1766667 | 117.6566667 |
| 34 | PPP2R2 | Cluster-73431.25844 | 29.56 | 35.95 | 27.84333333 |
| 35 | PPP2R3 | Cluster-73431.34770 | 23.4 | 32.14 | 26.17 |
|  |  | Cluster-73431.32181 | 28.89333333 | 84.36666667 | 60.49 |
|  |  | Cluster-73431.33566 | 21.39 | 38.12333333 | 35.86666667 |
|  |  | Cluster-73431.22392 | 143.81 | 78.02333333 | 75.4 |
| 36 | PPP2R5 | Cluster-73431.12728 | 9.373333333 | 14.78333333 | 12.75666667 |
|  |  | Cluster-73431.25180 | 17.95666667 | 46.81 | 32.57 |
|  |  | Cluster-73431.26159 | 47.99333333 | 38.21666667 | 38.04 |
|  |  | Cluster-73431.36524 | 8.463333333 | 16.44333333 | 11.77333333 |
|  |  | Cluster-73431.12402 | 6.556666667 | 2.78 | 2.123333333 |
|  |  | Cluster-73431.30562 | 6.626666667 | 9.636666667 | 9.59 |
| 37 | HBS1 | Cluster-73431.25313 | 19.09333333 | 16.77 | 14.73 |
| 38 | PELO | Cluster-73431.15257 | 30.26333333 | 22.74333333 | 24.72 |
