# Supplementary material for: Transcriptomic analysis reveals effects of fertilization towards growth and quality of Fritillariae thunbergii bulbus
Source: PLoS One. 2024 Sep 20;19(9):e0309978. doi: 10.1371/journal.pone.0309978 (PMC11414930; doi:10.1371/journal.pone.0309978)
Supplement: S7 Table — (DOCX) [file pone.0309978.s009.docx]

**S7 Table. Transcripts and FPKM of genes involved in ubiquitin mediated proteolysis (ko04120).**

| Number | Name | Gene ID | FPKM | | |
| --- | --- | --- | --- | --- | --- |
|  |  |  | RC | OF | PA |
| 1 | UBE1 | Cluster-73431.33560 | 54.78666667 | 117.78 | 83.07666667 |
|  |  | Cluster-73431.31467 | 35.93 | 69.26333333 | 50.28666667 |
|  |  | Cluster-73431.31213 | 102.4333333 | 162.7066667 | 112.8733333 |
| 2 | UBLE1B | Cluster-73431.24261 | 38.88 | 29.67333333 | 29.19 |
| 3 | UBE2C | Cluster-73431.29809 | 40.02333333 | 54.81 | 44.14333333 |
| 4 | UBE2D | Cluster-75167.0 | 0.580003333 | 0.396666667 | 2.19 |
|  |  | Cluster-73431.26923 | 1.246666667 | 0.396666667 | 0.273336667 |
|  |  | Cluster-73431.30555 | 556.6433333 | 371.87 | 430.9833333 |
| 5 | UBE2G1 | Cluster-73431.30818 | 1636.81 | 636.4833333 | 762.4766667 |
| 6 | UBE2I | Cluster-73431.16022 | 49.40666667 | 138.0966667 | 102.94 |
|  |  | Cluster-73431.32772 | 66.92 | 100.7933333 | 86.98333333 |
| 7 | UBE2J1 | Cluster-73431.30085 | 6.363333333 | 10.96666667 | 9.65 |
| 8 | UBE2J2 | Cluster-73431.23857 | 46.13666667 | 64.81 | 49.70333333 |
|  |  | Cluster-73431.30835 | 163.5066667 | 94.41 | 104.7566667 |
| 9 | UBE2N | Cluster-73431.34169 | 12.99333333 | 17.04666667 | 17.09 |
| 10 | UBE2O | Cluster-73738.0 | 1.016666667 | 0.15667 | 0.096673333 |
|  |  | Cluster-73431.34407 | 12.77333333 | 5.4 | 6.686666667 |
|  |  | Cluster-73431.23002 | 20.93666667 | 16.75333333 | 13.62 |
|  |  | Cluster-73431.32090 | 37.43 | 8.086666667 | 8.38 |
| 11 | UBE2S | Cluster-73431.33179 | 23.27666667 | 32.78666667 | 34.72333333 |
| 12 | UBE2W | Cluster-73431.25687 | 20.82 | 8.146666667 | 13.6 |
| 13 | UBE3B | Cluster-73431.11836 | 17.57333333 | 8.473333333 | 9.62 |
| 14 | UBE3C | Cluster-73431.35713 | 55.09333333 | 41.34666667 | 36.45 |
| 15 | TRIP12 | Cluster-74300.0 | 0.663333333 | 0.12667 | 0.726666667 |
| 16 | HUWE1 | Cluster-73431.28211 | 45.54 | 46.09666667 | 20.13666667 |
|  |  | Cluster-73431.34989 | 31.46333333 | 24.63 | 18.59666667 |
| 17 | STUB1 | Cluster-73431.42691 | 8.833333333 | 1.806666667 | 3.39 |
| 18 | PRPF19 | Cluster-73431.29432 | 132.15 | 55.33666667 | 69.95333333 |
| 19 | SIAH1 | Cluster-73431.9460 | 15.67666667 | 8.15 | 8.606666667 |
|  |  | Cluster-73431.17093 | 82.95333333 | 58.39 | 67.07666667 |
|  |  | Cluster-73431.9082 | 19.37666667 | 10.64 | 16.00666667 |
| 20 | RFWD2 | Cluster-73431.25402 | 23.43 | 15.13 | 16.50333333 |
| 21 | RCHY1 | Cluster-73431.24130 | 23.41666667 | 50.91666667 | 36.19 |
|  |  | Cluster-73431.25366 | 66.29333333 | 24.95333333 | 17.36666667 |
|  |  | Cluster-73431.25363 | 1.813333333 | 0.313333333 | 0.20667 |
|  |  | Cluster-73431.31581 | 107.8633333 | 63.60666667 | 66.57333333 |
|  |  | Cluster-73431.39127 | 27.79666667 | 10.57666667 | 11.47333333 |
|  |  | Cluster-73431.16179 | 5.096666667 | 2.03 | 5.916666667 |
| 22 | PIAS1 | Cluster-73431.33555 | 26.08333333 | 10.52 | 12.67 |
|  |  | Cluster-73431.42706 | 6.186666667 | 4.033333333 | 2.926666667 |
|  |  | Cluster-73431.17550 | 2.1 | 1.526666667 | 0.806666667 |
| 23 | RBX1 | Cluster-73431.31366 | 78.66 | 177.4833333 | 133.4733333 |
| 24 | CUL1 | Cluster-73431.24845 | 45.83333333 | 36.85333333 | 32.65666667 |
|  |  | Cluster-73431.20778 | 71.43333333 | 44.21 | 52.01333333 |
|  |  | Cluster-73431.51577 | 3.496666667 | 1.143333333 | 1.483333333 |
|  |  | Cluster-73431.4113 | 10.26333333 | 7.313333333 | 6.336666667 |
| 25 | SKP1 | Cluster-73431.29015 | 26.40333333 | 9.873333333 | 16.69333333 |
|  |  | Cluster-73431.20963 | 12.13333333 | 6.03 | 8.436666667 |
|  |  | Cluster-73431.32648 | 126.6133333 | 81.75 | 106.9766667 |
|  |  | Cluster-73431.3593 | 2.936666667 | 0.066673333 | 0.79 |
|  |  | Cluster-73431.46196 | 1.86667 | 5.46 | 0.570006667 |
| 26 | SKP2 | Cluster-73431.31849 | 12.86666667 | 9.086666667 | 4.976666667 |
|  |  | Cluster-73431.23495 | 8.433333333 | 2.46 | 1.843333333 |
| 27 | ELOC | Cluster-73431.15283 | 35.14333333 | 69.90333333 | 51.63333333 |
| 28 | CUL4 | Cluster-73431.31574 | 58.76 | 68.76333333 | 55.08666667 |
| 29 | DDB2 | Cluster-73431.16741 | 15.49333333 | 10.07 | 8.223333333 |
| 30 | ERCC8 | Cluster-73431.12324 | 12.84333333 | 10.70666667 | 9.573333333 |
| 31 | APC1 | Cluster-73431.25966 | 5.11 | 3.7 | 3.313333333 |
| 32 | APC3 | Cluster-73431.36356 | 10.69 | 4.983333333 | 5.1 |
|  |  | Cluster-73431.38261 | 58.21666667 | 28.49 | 35.53 |
|  |  | Cluster-73431.38262 | 28.56666667 | 19.69666667 | 18.70333333 |
|  |  | Cluster-41929.0 | 1.543333333 | 0.07667 | 0.070003333 |
| 33 | APC5 | Cluster-73431.22417 | 20.58333333 | 7.373333333 | 10.12666667 |
| 34 | APC7 | Cluster-73431.25834 | 41.35 | 24.08333333 | 26.71666667 |
| 35 | APC8 | Cluster-73431.14229 | 5.31 | 8.173333333 | 5.92 |
