# Supplementary material for: Transcriptomic analysis reveals effects of fertilization towards growth and quality of Fritillariae thunbergii bulbus
Source: PLoS One. 2024 Sep 20;19(9):e0309978. doi: 10.1371/journal.pone.0309978 (PMC11414930; doi:10.1371/journal.pone.0309978)
Supplement: S8 Table — (DOCX) [file pone.0309978.s010.docx]

**S8 Table. Transcripts and FPKM of genes involved in terpenoid backbone biosynthesis (ko00900).**

| Number | Name | Gene ID | FPKM | | |
| --- | --- | --- | --- | --- | --- |
|  |  |  | RC | OF | PA |
| 1 | ACAT | Cluster-73431.25563 | 88.67 | 125.4333333 | 106.1866667 |
|  |  | Cluster-35761.0 | 0.13667 | 0.00001 | 1.49 |
|  |  | Cluster-73431.30459 | 153.1033333 | 255.32 | 235.3133333 |
| 2 | HMGCS | Cluster-73431.26440 | 100.63 | 63.11333333 | 73.58 |
| 3 | HMGCR | Cluster-73431.34089 | 1.366666667 | 0.116666667 | 0.836666667 |
| 4 | E2.7.4.2 | Cluster-73431.19534 | 15.2 | 24.20666667 | 23.86666667 |
|  |  | Cluster-73431.50199 | 2.953333333 | 0.00001 | 0.00001 |
| 5 | mvd | Cluster-73431.42138 | 17.67666667 | 31.29666667 | 29.94 |
| 6 | dxs | Cluster-73431.14145 | 1.866673333 | 298.1666667 | 140.2 |
|  |  | Cluster-73431.16427 | 2.366673333 | 483.7733333 | 331.1166667 |
|  |  | Cluster-73431.16426 | 0.860003333 | 173.1566667 | 110.8566667 |
|  |  | Cluster-73431.15496 | 0.00001 | 12.32 | 14.61333333 |
|  |  | Cluster-73431.14781 | 2.32 | 126.19 | 88.65666667 |
|  |  | Cluster-73431.14782 | 2.023336667 | 12.24333333 | 27.92333333 |
|  |  | Cluster-73431.41369 | 0.00001 | 21.48333333 | 46.43333333 |
|  |  | Cluster-73431.41367 | 2.196666667 | 54.08 | 78.72666667 |
|  |  | Cluster-73431.15772 | 0.526673333 | 10.02 | 13.52333333 |
|  |  | Cluster-73431.41572 | 0.483336667 | 55.87 | 34.20666667 |
|  |  | Cluster-73431.14045 | 0.973336667 | 21.43666667 | 8.446666667 |
|  |  | Cluster-73431.41616 | 2.150003333 | 176.47 | 128.65 |
|  |  | Cluster-73431.16499 | 3.493333333 | 751.9933333 | 387.35 |
|  |  | Cluster-73431.14810 | 0.00001 | 9.19 | 8.866666667 |
|  |  | Cluster-73431.42169 | 0.316673333 | 140.6 | 141.14 |
|  |  | Cluster-73431.41891 | 0.00001 | 101.8966667 | 13.75666667 |
|  |  | Cluster-73431.41405 | 2.28 | 386.6866667 | 249.9533333 |
|  |  | Cluster-73431.41407 | 0.160006667 | 71.01 | 36.94333333 |
|  |  | Cluster-73431.41406 | 3.616666667 | 328.17 | 234.6666667 |
|  |  | Cluster-73431.41650 | 0.056673333 | 1.396666667 | 2.976666667 |
|  |  | Cluster-73431.41409 | 6.526666667 | 837.6133333 | 574.3966667 |
| 7 | dxr | Cluster-73431.15565 | 1.216666667 | 1.326666667 | 2.563333333 |
|  |  | Cluster-73431.33807 | 31.35666667 | 473.99 | 324.52 |
| 8 | ispD | Cluster-73431.29964 | 25.08333333 | 55.82333333 | 50.77333333 |
| 9 | ispE | Cluster-73431.25450 | 65.28 | 94.75333333 | 96.41 |
| 10 | ispF | Cluster-73431.23573 | 14.12 | 36.17 | 32.51 |
| 11 | gcpE | Cluster-73431.35647 | 63.11 | 463.5066667 | 312.9533333 |
|  |  | Cluster-73431.35648 | 20.7 | 114.0133333 | 98.99666667 |
| 12 | ispH | Cluster-73431.32958 | 205.5266667 | 788.3466667 | 680.44 |
| 13 | ispS | Cluster-73431.41306 | 6.093333333 | 0.573333333 | 3.263333333 |
|  |  | Cluster-73431.41305 | 5.86 | 0.623333333 | 5.953333333 |
|  |  | Cluster-73431.41261 | 24.70333333 | 1945.886667 | 1319.29 |
|  |  | Cluster-73431.12400 | 1.28 | 1.25 | 3.606666667 |
| 14 | FDPS | Cluster-73431.30524 | 116.3866667 | 92.34666667 | 117.4633333 |
| 15 | GGPS | Cluster-73431.31286 | 90.36333333 | 1016.646667 | 801.9933333 |
|  |  | Cluster-73431.34565 | 41.33333333 | 470.1566667 | 404.49 |
|  |  | Cluster-73431.16306 | 3.220003333 | 4.436666667 | 28.13 |
|  |  | Cluster-58937.1 | 14.67666667 | 3.23 | 4.153333333 |
|  |  | Cluster-73431.41251 | 7.45 | 809.5233333 | 789.7366667 |
|  |  | Cluster-73431.34257 | 40.37333333 | 248.1433333 | 500.9166667 |
|  |  | Cluster-73431.29675 | 0.00001 | 2.776666667 | 3.253333333 |
|  |  | Cluster-73431.18131 | 21.01333333 | 57.24 | 35.84333333 |
|  |  | Cluster-73431.41249 | 0.693333333 | 311.64 | 120.2166667 |
|  |  | Cluster-73431.24439 | 3.993333333 | 4.256666667 | 28.24 |
|  |  | Cluster-73431.29287 | 14.27 | 149.8866667 | 164.7233333 |
|  |  | Cluster-73431.29289 | 30.28 | 354.33 | 411.9033333 |
| 16 | GPS | Cluster-73431.39604 | 23.81333333 | 33.84666667 | 25.39 |
| 17 | FLDH | Cluster-73431.20713 | 9.056666667 | 11.87 | 12.57666667 |
| 18 | PCYOX1 | Cluster-73431.43084 | 11.95666667 | 22.07333333 | 19.93 |
| 19 | PCME | Cluster-73431.30559 | 69.52333333 | 79.96 | 68.4 |
|  |  | Cluster-73431.11650 | 2.526666667 | 6.87 | 4.936666667 |
| 20 | FNTB | Cluster-73431.25223 | 37.94333333 | 24.68 | 27.64333333 |
| 21 | chIP | Cluster-73431.23194 | 12.95666667 | 23.81 | 15.01333333 |
|  |  | Cluster-73431.23193 | 38.77 | 45.23666667 | 18.27333333 |
