# Supplementary material for: Transcriptomic analysis reveals effects of fertilization towards growth and quality of Fritillariae thunbergii bulbus
Source: PLoS One. 2024 Sep 20;19(9):e0309978. doi: 10.1371/journal.pone.0309978 (PMC11414930; doi:10.1371/journal.pone.0309978)
Supplement: S9 Table — (DOCX) [file pone.0309978.s011.docx]

**S9 Table. Transcripts and FPKM of genes involved in steroid biosynthesis (ko00100).**

| Number | Name | Gene ID | FPKM | | |
| --- | --- | --- | --- | --- | --- |
|  |  |  | RC | OF | PA |
| 1 | SQS | Cluster-73431.27722 | 143.6066667 | 105.6233333 | 117.3666667 |
| 2 | DHCR24 | Cluster-73431.31917 | 27.91666667 | 38.08666667 | 29.81 |
| 3 | NSDHL | Cluster-73431.16347 | 27.53 | 38.96333333 | 31.74666667 |
|  |  | Cluster-73431.15956 | 18.87666667 | 17.15333333 | 16.52666667 |
|  |  | Cluster-73431.12683 | 3.373333333 | 7.633333333 | 5.97 |
| 4 | EBP | Cluster-73431.25385 | 10.7 | 17.98 | 18.57333333 |
| 5 | SMT1 | Cluster-73431.16359 | 18.61333333 | 50.59666667 | 55.61 |
|  |  | Cluster-73431.21605 | 22.23 | 9.706666667 | 24.95 |
|  |  | Cluster-73431.21604 | 5.43 | 2.61 | 7.403333333 |
| 6 | SC5DL | Cluster-73431.27869 | 88.11666667 | 54.89333333 | 77.08666667 |
| 7 | CAS1 | Cluster-73431.5702 | 2.176666667 | 0.6 | 0.373333333 |
| 8 | SMO1 | Cluster-73431.26327 | 46.16333333 | 98.33 | 85.79333333 |
| 9 | SMO2 | Cluster-73431.31700 | 68.02 | 48.82666667 | 42.83666667 |
|  |  | Cluster-4451.0 | 3.153333333 | 0.00001 | 0.00001 |
|  |  | Cluster-73431.322 | 1.57 | 2.333333333 | 2.996666667 |
| 10 | SMT2 | Cluster-73431.28943 | 46.28333333 | 131.5566667 | 114.4666667 |
| 11 | DHCR7 | Cluster-73431.30024 | 630.72 | 427.12 | 472.8066667 |
| 12 | CYP710A | Cluster-73431.16268 | 7.846666667 | 11.1 | 27.74 |
| 13 | LIPA | Cluster-73431.25446 | 51.25333333 | 22.38333333 | 28.74 |
| 14 | TGL4 | Cluster-73431.26472 | 16.50333333 | 8.083333333 | 12.55 |
| 15 | SQLE | Cluster-73431.6662 | 1.21 | 2.04 | 2.176666667 |
