# Supplementary material for: Transcriptomic analysis reveals effects of fertilization towards growth and quality of Fritillariae thunbergii bulbus
Source: PLoS One. 2024 Sep 20;19(9):e0309978. doi: 10.1371/journal.pone.0309978 (PMC11414930; doi:10.1371/journal.pone.0309978)
Supplement: Attachment — Submitted filename: Response.docx [file pone.0309978.s013.docx]

Dear Editor and Reviewers:

Thanks for your letter and the reviewers' comments concerning our manuscript, "**Transcriptomic analysis reveals effects of fertilization towards growth and quality of *Fritillariae thunbergii* bulbus**" (Manuscript ID: PONE-D-24-13936). Those comments are all valuable and helpful for revising and improving our paper. We have studied all comments carefully and have made conscientious corrections. Our response is given in standard font, and changes to the manuscript are given in red text. The responses to the reviewers' comments are as follows:

Response to the reviewers:

Reviewer 1

We feel great thanks for your professional review work on our article. As you are concerned, there are several problems that need to be addressed. According to your nice suggestions, we have made extensive corrections to our previous draft, the detailed corrections are listed below.

1. The Abstract is lacking key results like the yield and alkaloids content obtained from the best fertilizer.

Answer: Line 25-27, the yield and alkaloids content of “The combined results of HPLC-ELSD detection and yield statistics showed that the *F. thunbergii* with OF application was the best, with the content of peimine and peiminine reaching 0.0603% and 0.0502%, respectively. In addition, the yield was 2.70 kg/m². ” was added.

2. In the introduction, it is preferable to state the hypothesis tested rather than the objectives.

Answer: Line 71-77, and Line 82-84, based on existing literatures on the correlation between altitude and secondary metabolic yield of medicinal plants, and the application to *in vitro* cultivation of Himalayan *Fritillaria* species, it is speculated that different fertilization methods may be related to the alkaloid content and yield of *Fritillaria.*

3. In the methods section, the authors should justify why they use only one cultiver rather than using many. the quantity and justification of the quantity of each of the fertiliser used for the study. what was the growing conditions of the plants. Alkaloids content was evaluated of plant tissue of how old? You should use AOVA test with mean separation as a statistical means. Also provide The software used for clustering transcriptome data.

Answer: To control variables, we selected the same batch of *Fritillariae thunbergii* bulbs for different fertilization conditions to prevent the influence of different varieties on their alkaloid contents. Among them, ‘Zhebei 1’ has a wide range of applications, which is more representative and practical.

The growth environment of *Fritillariae thunbergii* bulbs in the three experimental fields was the same natural environment, with the same altitude 600 m.

Three year old bulbs of *Fritillariae thunbergii* were used in this experiment.

Line 173-174, and Line 194-196, we conducted One-way ANOVA of alkaloid content of different fertilization conditions using SPSS (25.0.0).

Line 161-162, “Furthermore, the clustered expression pattern was drawn through R (Version 3.0.3), Package ggplot2 and Package pheatmap.” were added.

Additionally, Line 184-193, we have provided a more detailed description of the experimental results.

4. There is no standard deviation?- yield

Answer: Thank you for your reminder. Line 93-94, *Fritillaria thunbergii* bulb yield was determined by harvesting the plants in an area of 2 m^2^ in each plot. We collected bulbs from a whole experimental field and calculated the yield.

Reviewer 2

The manuscript entitled "Transcriptomic analysis reveals effects of fertilization towards growth and quality of Fritillariae thunbergii bulbus" provides valuable insights into optimizing the cultivation conditions of Fritillariae thunbergii (FTB), a traditional Chinese medicine renowned for its medicinal properties. The study investigates the impact of different fertilizers on the yield and quality of FTB, focusing on raw chicken manure (RC), organic fertilizer (OF), and plant ash (PA). Through a combination of HPLC-ELSD detection and yield statistics, OF application emerged as the most effective fertilizer. Transcriptome analysis uncovered the up-regulation of the ABA signaling pathway, potentially enhancing bulb yield, along with the identification of key genes associated with steroidal alkaloid accumulation. However, the manuscript requires minor revisions.

Answer: Thank you for your valuable comments, revisions have been made to improve the readability of the present manuscript. Detailed changes and corresponding answers have been displayed below.

1. Replication information regarding biological and technical replicates should be provided.

Answer: Line 95-96, “ all the bulbs were collected biological replicates to randomly select different regions in the north, south, east, and west of the same production area” was added. Line 99-100, and Line 112-113, the number biological replicates has been explained.

2. Transcription factor/gene names should be italicized throughout the manuscript.

Answer: We were really sorry for our careless mistakes. Thank you for noticing. Transcription factor/gene names were corrected to Italic format.

3. The methodology section should include NCBI SRA submission information.

Answer: Line 175-177, “**Data avaliability:** The raw data of RNA-Seq have been submitted to the the NCBI Sequence Read Archive (SRA) database (Accession: PRJNA950562).” was added.

4. The introduction and discussion sections would benefit from the inclusion of references such as (Kumar, V., Sharma, S., Kumar, P. (2024). Insight into the Genetics and Genomics Studies of the Fritillaria Species. In: Gahlaut, V., Jaiswal, V. (eds) Genetics and Genomics of High-Altitude Crops. Springer, Singapore. https://doi.org/10.1007/978-981-99-9175-4_4;https://doi.org/10.1016/j.indcrop.2023.117541; Kumar, V., Kumar, P., Bhargava, B. et al. Transcriptomic and Metabolomic Reprogramming to Explore the High-Altitude Adaptation of Medicinal Plants: A Review. J Plant Growth Regul 42, 7315–7329 (2023). https://doi.org/10.1007/s00344-023-11018-8; Kapoor, B., Kumar, A. & Kumar, P. Transcriptome repository of North-Western Himalayan endangered medicinal herbs: a paramount approach illuminating molecular perspective of phytoactive molecules and secondary metabolism. Mol Genet Genomics 296, 1177–1202 (2021). https://doi.org/10.1007/s00438-021-01821-x) to enrich the context and support the findings. Overall, these adjustments will enhance the clarity and completeness of the manuscript, contributing to its scientific significance.

Answer: We sincerely appreciate the valuable comments. We have checked the literature carefully and added the four references on 11 (Line 58-60), 15 (Line 71-72), 16 (Line 71-72) and 17 (Line 72-73) into the Introduction part in the revised manuscript.

Thank you very much for your attention and time. Look forward to hearing from you.

Yours sincerely,

Corresponding author:

Name: Zhuoheng Zhong

E-mail: zhongzhh@zstu.edu.cn
